# Quantitative 3-Dimensional Imaging of Murine Neointimal and Atherosclerotic Lesions by Optical Projection Tomography

**DOI:** 10.1371/journal.pone.0016906

**Published:** 2011-02-17

**Authors:** Nicholas S. Kirkby, Lucinda Low, Jonathan R. Seckl, Brian R. Walker, David J. Webb, Patrick W. F. Hadoke

**Affiliations:** Centre for Cardiovascular Science, University of Edinburgh, Edinburgh, United Kingdom; Universität Würzburg, Germany

## Abstract

**Objective:**

Traditional methods for the analysis of vascular lesion formation are labour intensive to perform - restricting study to ‘snapshots’ within each vessel. This study was undertaken to determine the suitability of optical projection tomographic (OPT) imaging for the 3-dimensional representation and quantification of intimal lesions in mouse arteries.

**Methods and Results:**

Vascular injury was induced by wire-insertion or ligation of the mouse femoral artery or administration of an atherogenic diet to apoE-deficient mice. Lesion formation was examined by OPT imaging of autofluorescent emission. Lesions could be clearly identified and distinguished from the underlying vascular wall. Planimetric measurements of lesion area correlated well with those made from histological sections subsequently produced from the same vessels (wire-injury: R^2^ = 0.92; ligation-injury: R^2^ = 0.89; atherosclerosis: R^2^ = 0.85), confirming both the accuracy of this methodology and its non-destructive nature. It was also possible to record volumetric measurements of lesion and lumen and these were highly reproducible between scans (coefficient of variation = 5.36%, 11.39% and 4.79% for wire- and ligation-injury and atherosclerosis, respectively).

**Conclusions:**

These data demonstrate the eminent suitability of OPT for imaging of atherosclerotic and neointimal lesion formation, providing a much needed means for the routine 3-dimensional analysis of vascular morphology in studies of this type.

## Introduction

The formation of vascular lesions in response to acute or chronic injury to the arterial wall defines atherosclerosis and post-interventional restenosis, conditions that contribute greatly to cardiovascular morbidity and mortality[1]. Understanding of the processes that lead to such lesion formation has been advanced considerably by exploiting murine models of acute vascular injury and atherosclerosis that are amenable to genetic manipulation[2]. Despite these advances, *ex vivo* identification and quantification of vascular lesions typically relies on 2-dimensional histological analysis. This is time consuming and provides only limited information on lesion volume. In particular, lesion burden in an artery is commonly assessed by measurement of cross-sectional lesion area, either at randomly selected sites along its profile or at the site of maximum occlusion, providing an imperfect analysis of overall lesion burden.

Whole-mount 3-dimensional imaging technology should provide a solution to this problem, yet surprisingly few suitable approaches have been described. This reflects the scale of the murine vasculature – too large for microscopic techniques such as single-photon confocal microscopy but too small for techniques derived from the clinic, including magnetic resonance imaging (MRI)[3] and computed tomography (CT)[4]. Indeed, whilst both *ex vivo* MRI and micro CT have been applied to the study of murine atherosclerosis, they offer limited resolution, even in relatively large arteries and require long acquisition times limiting throughput [3], [5]. Several newer optical imaging modalities have been described in attempt to span this region of scale poorly served by traditional systems. For example, optical coherence tomography [6] and photo-acoustic tomography [7] offer tissue penetration depths of 1–3 mm and imaging of optical scattering and absorbance, and do so at relatively high resolution.

Another such technique is optical projection tomography (OPT). Originally conceived for the study of mouse embryos, OPT is able to image biological specimens approximately ∼0.3 to 10 mm in diameter, using two image modes [8]. Transmission imaging records the opacity of a semi-translucent sample to polychromatic visible light and therefore primarily describes its pattern of absorbance. This may reflect the presence of pigments such as hemoglobin or of particles resistant to optical clearing, and can often be used to distinguish anatomical structures. Emission imaging records the ability of endogenous (e.g. collagen, elastin, NADPH, certain amino acids) and exogenous fluorophores contained within that sample to emit light upon excitation at specific wavelengths. This imaging mode may also describe anatomical structure because the individual tissue components making up a sample can differ in the type and density of autofluorescent species present. Further, through the use of fluorescent reporters the distribution of immunoreactivity or gene expression may be determined [9]. For both imaging modes, light (transmitted or emitted) is focused to a charge coupled device by conventional microscope optics, and images captured at multiple increments of rotation (typically 400 images at 0.9° increments). From these, the 3-dimensional volume can be calculated by standard tomographic reconstruction methods such as filtered back-projection or iterative reconstruction. A full description of the method can be found elsewhere [8], [9].

Since its introduction, the application of OPT has been broadened considerably, for example, having being adapted to the study of β-cells in the diabetic mouse pancreas, and the development of neuronal structures in human tissue samples[10]. Surprisingly given the need for such methodology, the suitability of OPT imaging for the 3-dimensional assessment of morphology in vascular tissues from adult animals has not been determined.

We addressed the proposal that OPT could be used as a rapid and cost-effective method to produce quantifiable 3-dimensional images of intimal lesions within murine arteries. The suitability of this technique was assessed using three commonly used models of murine vascular injury: femoral artery wire-injury and ligation models of neointimal hyperplasia and the apolipoprotein E-deficient (apoE^-/-^) mouse model of atherosclerosis.

## Methods

### Induction of neointima formation

All animal experiments were performed in accordance with the Animals (Scientific Procedures) Act (UK), 1986 and approved by the University of Edinburgh ethical review committee (PPL 60/3867). Surgical procedures were carried out under isoflurane anaesthesia and with buprenorphine post-operative analgesia. Acute vascular injury was performed in male, 12 week old C57Bl6/J mice (Harlan, UK). Wire-injury to the femoral artery was performed by insertion of a 0.014” diameter wire into the left femoral artery, previously described[11]. Ligation-injury was performed by occlusion of the right femoral artery of the same mice with a 5-0 silk ligature across the femoropopliteal bifurcation, analogous to the carotid artery ligation-injury model[12]. Animals were allowed to recover for 28 days before sacrifice by transcardiac perfusion fixation.

### Induction of atherosclerosis

Atherosclerotic lesion formation was induced in male, 6 week old ApoE-null mice (bred in house) by feeding a Western diet (0.2% cholesterol; Research Diets, USA) for 12 weeks. At the end of this period, animals were killed by perfusion fixation.

### OPT scanning and quantification

For OPT imaging, isolated vessels were embedded in 1.5% low melting point agarose (Invitrogen, UK), dehydrated in methanol (100%; 24 hours) and optically cleared in benzyl alcohol:benzyl benzoate (1∶2 v/v; 24 hours). Vessels were imaged using a Bioptonics 3001 OPT tomograph. All studies on injured arteries were performed using emission imaging, after UV illumination (425 nm excitation filter with 40 nm band pass; 475 nm long pass emission filter; 1.048Mpixel scanning resolution). For each vessel type, a magnification was chosen to provide the smallest voxel size whilst allowing the entire region of interest to be covered. This resulted in voxel sizes of 216, 64 and 166 µm^3^ respectively for wire- and ligation-injured femoral arteries and atherosclerotic aortic arches. For each vessel, exposure time was adjusted to maximise the dynamic range of the resulting image and was approximately 400, 800 and 1000ms per projection for wire- and ligation-injured femoral arteries and atherosclerotic aortic arches, respectively. Raw data (400 projections per scan at 0.9° increments) was subject to Hamming-filtered back-projection using NRecon software (Skyscan, Belgium). Quantification was performed using CTan software (Skyscan, Belgium). Briefly, lesion and lumen volumes were segmented by semi-automated tracing of the internal elastic lamina and subsequent grey-level thresholding to distinguish neointima from lumen. (See **Methods S1** for a step-by-step protocol).

### Histology

After tomographic scanning, vessels were immersed in methanol for 24 hours, trimmed of excess agarose and processed to paraffin wax. Histological sections (3–4 µm thick) underwent ‘United States Trichrome’ (UST) staining to highlight morphology[13] or picro-sirius red to highlight collagen. Measurements of lesion area (defined as the area between the luminal border and internal elastic lamina) were recorded from photomicrographs of UST-stained sections using Photoshop CS4 Extended software (Adobe Inc, USA).

### Immunohistochemistry

Immunohistochemistry was performed for α-smooth muscle actin (αSMA) and Mac2. Briefly, rehydrated sections were incubated with mouse anti-αSMA (Sigma, UK; 1/400 dilution; 30 mins) or rat anti-Mac2 primary antibodies (Cederlane Inc, USA; 1/6000 dilution; overnight) before treatment with goat anti-mouse IgG (Vector Labs, UK; 1/400 dilution; 30 mins) or goat anti-rat IgG secondary antibodies (Vector Labs, UK; 1/200 dilution; 30 mins), respectively. Sections were then treated with streptavidin-conjugated peroxidase (Extravidin-Peroxidase; Sigma, UK; 1/400 dilution; 30 mins) and the stain developed by application of 3,3-diaminobenzidine.

### Statistics and data analysis

OPT slices corresponding to histological sections were identified based on the distance of each from a fixed reference point within the sample (the femoropopliteal bifurcation, the ligation and the root of the brachiocephalic artery, respectively, for wire- and ligation-injured femoral arteries and atherosclerotic aortic arches). As z-axis distances can only be approximated from serial sections, visual cues, such as the position of arterial branches and vessel conformation were, in some cases, used to aid selection of appropriate image pairs. Measurements of lesion areas from OPT and histological sections were then compared by linear regression. The deviation of the slope from 1.0 was determined by F-test. Precision was assessed by calculation of the coefficient of variation between lesion volume measurements made from four independent scans/reconstructions of the same vessels.

Additional methodological detail is provided in **Methods S2**.

## Results

### Transmission and emission imaging of isolated arteries

To determine the suitability of OPT for the study of isolated murine blood vessels, healthy femoral arteries (n = 5) were each scanned twice - for visible light transmittance, and for fluorescent emission. In the transmission channel, the opacity of these tissues was so low as to preclude the capture and reconstruction of useful data. In emission channels, however, femoral arteries were seen to autofluoresce strongly, with the greatest signal following excitation at 405–445 nm, corresponding to a 410 nm excitation peak for elastin[14]. In reconstructed 2-dimensional slices of arteries imaged in this way, the medial layer was clearly distinguished from the adventitia and lumen by its greater fluorescent signal (not shown). Fluorescent emission imaging was thus adopted for all further investigations.

### Imaging Intimal Lesions

**Figure 1:**
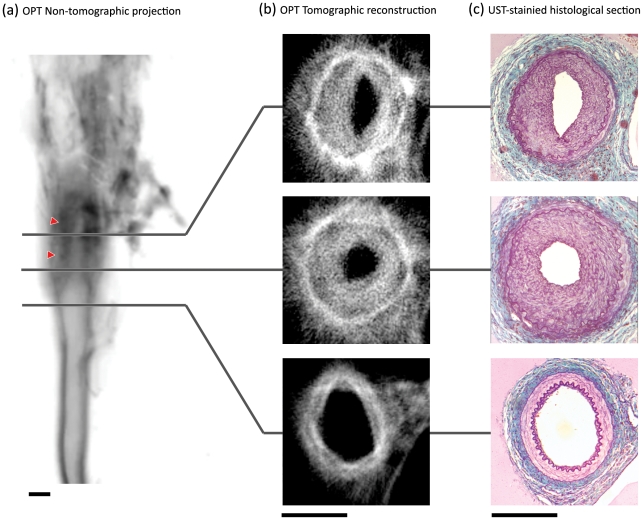
Identification of neointimal lesions in the ligation-injured mouse femoral artery. In non-tomographic fluorescent emission images of a ligation-injured femoral artery (a) neointimal thickening is clearly visible (red arrowheads). Image has been inverted for clarity (darker regions reflect stronger emission). In tomographic reconstructions (b), all layers of the vessel wall can be identified. Reconstructions strongly resemble US trichrome-stained histological sections of the same vessels (c). Scale bars in (a–c) are 200 µm.

To establish whether OPT imaging could characterise neointimal lesion formation, mouse femoral arteries were subject to wire- (n = 6) or ligation- (n = 5) injury. Following either insult, neointimal thickening could be seen clearly in non-tomographic emission projections (**Figure 1a**). In reconstructed 2-dimensional slices, concentric neointimal lesions were obvious and could be distinguished from the media by their weaker emission (**Figure 1b** **, Figure S1**).

**Figure 2:**
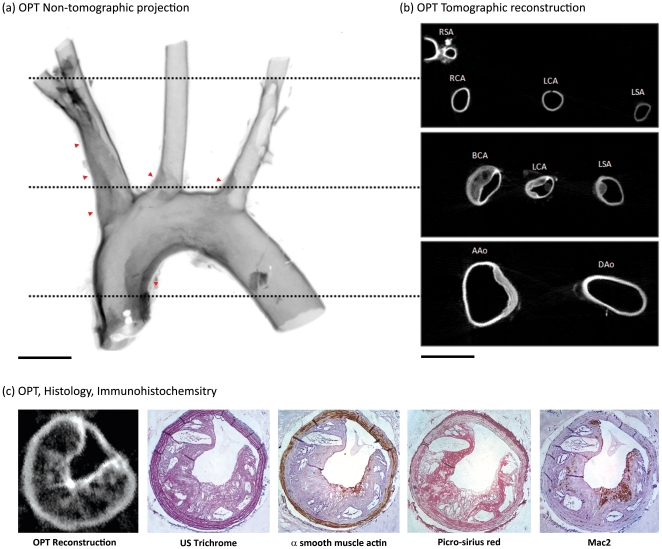
Identification of atherosclerotic lesion formation in the aortic arch of apoE^-/-^ mice. In non-tomographic fluorescent emission images of the aortic arch of atherosclerotic mice (a), lesion formation (red arrowheads) is apparent with the expected distribution. Image has been inverted for clarity (darker regions reflect stronger emission). In cross-sectional reconstructions, lesions are visible with the same distribution (b) and possess strong resemblance to histological sections of the same vessels (c). Successful analysis of the brachiocephalic artery by standard (immuno)-histochemical procedures further emphasises the non-destructive nature of this technique (c). Scale bars in (a–b) are 1 mm. RSA, right subclavian artery; RCA, right carotid artery; LCA, left carotid artery; LSA, left subclavian artery; BCA, brachiocephalic artery; AAo, ascending aorta; DAo, descending aorta.

To extend the possible application of this technique to atherosclerotic lesions in large conduit arteries, the aortic arch from apoE^-/-^ mice was also examined (n = 8). In OPT emission images of atherosclerotic aortic arches, lesion formation was again clearly evident with the expected anatomical distribution comprising lesions in the lesser curvature of the arch, in the brachiocephalic artery and at the origins of the left carotid and left subclavian arteries (**Figure 2a**). In cross-section, these lesions typically possessed an eccentric morphology and could be distinguished from media and lumen (**Figure 2b,c** **, Figure S2**).

### 2-dimensional validation and quantification

**Figure 3:**
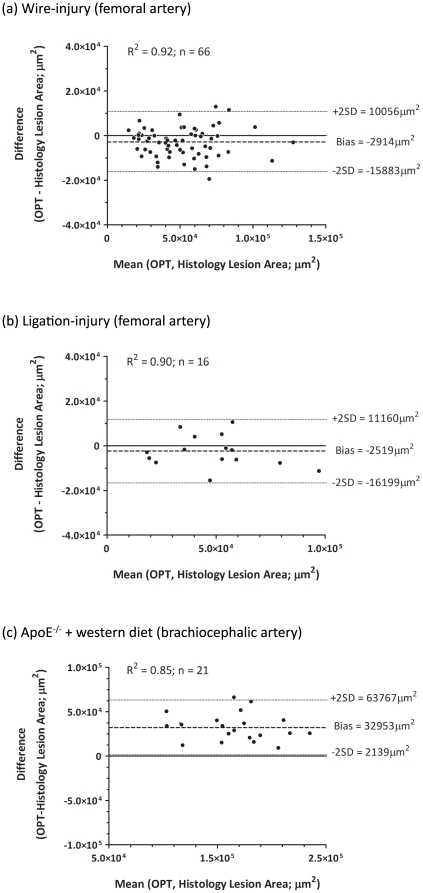
Comparison of planimetric measurements of lesion size between OPT reconstructions and histological sections. Planimetric measurements of lesion size recorded from OPT and histology image sets correlate strongly for all lesion types; Bland-Altman analysis indicates that this is unbiased for wire- (a) and ligation-injured femoral arteries (b) and that OPT measurements have a consistent positive bias in atherosclerotic brachiocephalic arteries (c).

To validate the interpretation of OPT reconstructions following tomographic imaging, the same arteries were processed for histological examination. For both neointimal (**Figure 1c**) and atherosclerotic lesions (**Figure 2c**), histological sections and corresponding OPT reconstructions were strikingly similar. We compared planimetric measurements of lesion area obtained by each method. For wire- and ligation-induced neointimal lesions and atherosclerotic plaques, these correlated strongly by linear regression (R^2^ = 0.92, R^2^ = 0.89 and R^2^ = 0.85, respectively) and the slope of these relationships did not differ from 1 (p = 0.49, p = 0.58 and p = 0.88, respectively). Bland-Altman analysis indicated no obvious bias in measurements of neointimal lesions induced by either injury (**Figure 3a,b**) but a small positive bias in OPT-derived measurements of lesion size in the brachiocephalic artery (**Figure 3c**), possibly reflecting shrinkage of these larger vessels during histological processing. Importantly, however, this bias was independent of lesion size indicating that measurements made in this way are comparable within and between vessels. We also confirmed that the OPT imaging procedure did not compromise subsequent analysis of lesion composition. In vessels previously subjected to OPT scanning, (immuno)staining for markers of smooth muscle (α-smooth muscle actin), macrophages (Mac2) and collagen (picro-sirius red) all occurred with the expected intensity and distribution (**Figures 2c**).

### 3-dimensional quantification and reproducibility

**Figure 4:**
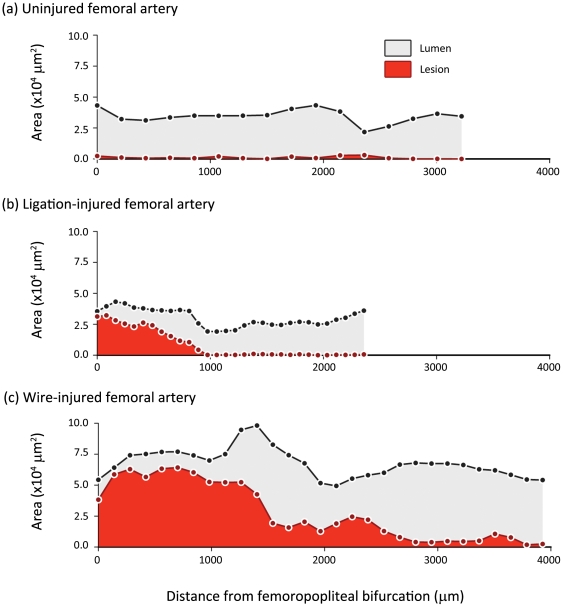
Longitudinal profiles of lesion and lumen cross-sectional area in un-injured, ligation- and wire-injured femoral arteries. When cross-sectional area of lesion (red) and lumen (grey) are plotted against distance along the axial length of the femoral artery, the difference in extent of lesion formation between ligation- (b) and wire-injury models (c) is apparent. In both cases, the variability in lesion size that occurs along the length of each vessel is also highlighted.

Whilst 2-dimensional comparisons of OPT and histology provide crucial validation, the greater potential of OPT is in the consideration of 3-dimensional parameters. We established protocols for the volumetric quantification of lesions by OPT. Using these methods, we were able to record lesion volumes in wire- (0.1100±0.0091 mm^3^; n = 6) and ligation-injured femoral arteries (0.0200±0.0089 mm^3^; n = 5) and in atherosclerotic brachiocephalic arteries (0.18±0.018 mm^3^; n = 8). As would be expected from the degree of injury, lesions in wire-injured vessels were significantly greater (p<0.0001 by unpaired t-test) than those in ligation-injured vessels. Coefficients of variation (5.36%, 11.39% and 4.79%, respectively, n = 4) indicated that for all lesion types, measurements were highly reproducible. These data were also expressed as profiles of lesion burden along the length of the vessel (**Figure 4**) and rendered for dynamic, qualitative evaluation (see **Figures S1–S3**), in both cases, highlighting the uneven distribution of lesion formation occurring in injured vessels.

## Discussion

We have demonstrated, for the first time, the eminent suitability of OPT for the assessment of vascular structure in isolated arteries. Using this technique, 3-dimensional images describing the autofluorescent emission of the specimen were used to provide anatomical information on lesion size and distribution, as well as reproducible quantification of both the lesion and the residual lumen. This was achieved in vessels spanning the commonly studied size range in mice, and should be equally suitable for analysis of vascular lesions from other species, including small-to-medium sized human vessels. This methodology represents a considerable advance over traditional histological and, alternative 3-dimensional imaging techniques for the analysis of intimal lesion formation in small arteries.

Application of OPT to three widely-used models of arterial lesion development in the mouse, was designed to assess its potential for imaging atherosclerotic and proliferative lesions in large (aorta) and small (femoral) arteries. Importantly, these vessels define the required resolution for studies of this type - the aorta being the largest artery, and the femoral artery, which has a lumen diameter of ∼200–250 µm[15], being amongst the smallest vessels in which studies of vascular lesion formation are commonly performed. The striking similarities of OPT-generated images and those obtained from subsequent histological analysis of the same samples confirmed the suitability of the technique in both vessels. Moreover, we were able to quantitatively validate this similarity. The consistency of cross-sectional area measurements obtained from OPT with those obtained by planimetric analysis of the corresponding histologically-stained sections (the current gold standard) confirmed the accuracy of the former. Further, because each OPT data set is composed of many hundreds of cross-sectional scan lines, using OPT it was simple to generate profiles of lesion area along the injured vessel. The advantage of this was exemplified by the comparison of lesions induced by wire-injury[11] with those caused by arterial ligation[12]. This demonstrated that whilst cross-sectional narrowing caused by wire-injury and ligation were broadly similar close to the origin of the injury, the lesions resulting from insertion of a wire extended a far greater distance along the arterial wall. Generating comparable data from histological sections would have been expensive and extremely labour intensive.

Whilst these advantages alone might be sufficient to warrant adoption of OPT as the analytical method of choice; the real strength of the technique is conferred by the ability to quantify lesion volume. Measurements of lesion volume provide a more representative of the total lesion burden in an artery[3], and are therefore not subject to the bias and error introduced by selection of particular portions of the vessel for analysis. This is again emphasised in the current investigation by longitudinal profiles of lesion formation produced from OPT images, which indicate just how much lesion size can vary over short lengths of injured vessel. Whilst these advantages may be shared with other 3D imaging modalities, in comparison to previously reported MRI and micro-CT *ex vivo* examinations of small arteries, OPT appears to produce images of comparable or superior quality, yet with much shorter imaging times and less expense. Indeed, despite preparation of samples for OPT imaging taking several days, little labour is required on each occasion. As such, many vessels can be prepared in parallel and data acquired in one session, thereby providing a relatively high throughput method of imaging arterial lesions and not requiring prolonged use of the imaging device.

In addition to direct 2- and 3-dimensional quantification of vessel morphology from OPT images, because this technique is non-destructive, it can be considered complementary to traditional analysis. Rather than laboriously sectioning, staining and analysing long lengths of lesions to find sites of interest for immunohistochemical examination, OPT images can be used to quickly identify such regions for selective sectioning and subsequent, in-depth analysis using standard histology and immunohistochemistry. Thus, OPT imaging of vascular lesions can be easily adopted into existing workflows to accelerate traditional methods of analysis and provide volumetric information from the same tissues.

There are inevitably limitations of OPT-based analysis of arterial lesions that must be considered. First, although image quality is comparable or superior to that of other tomographic imaging systems, it is noticeably inferior to microscopic imaging techniques (which can, of course, only be performed on smaller segments). Several refinements to the back-projection algorithms used to reconstruct OPT data sets have been proposed which may in future, further improve the quality of images produced by this method[16], [17]. Second, the reagents used to prepare samples for OPT imaging might potentially alter certain aspects of the specimen. Perhaps the biggest potential source of artefacts resulting from the OPT procedure described here is the choice of a lipophilic agent (benzyl alcohol/benzyl benzoate) for optical clearing, which is likely to, for example, remove lipid pools from atherosclerotic plaques, and requires prior dehydration, which may cause shrinkage of tissue. In contrast to hydrophilic clearing agents such as glycerol, however, benzyl alcohol/benzyl benzoate is reported to cause minimal deformation of tissue morphology [18]. It is also important to consider that similar dehydration and lipid removal steps are otherwise required in the preparation of paraffin-embedded tissue sections. Further, although we have not directly compared tissues subject to both OPT imaging and histological analysis, with those only subject to the latter, we feel that the appearance and immunoreactivity of lesions in these models are subjectively similar to those we have previously observed in directly prepared histological sections. Beyond these rather general limitations, those more specific to the methodology presented here appear relatively minor. Ironically, perhaps the most obvious of these derives from the strong autofluorescence of arterial tissue, which enables such clear anatomical imaging. We have found this to be resistant to bleaching by previously set out methods[19], and as such, it may restrict the use of fluorescent probes to assess RNA and protein distribution patterns.

Much potential for further development of this technique exists, particularly with regard to tracking the 3-dimensional arrangement of key cells and signalling factors involved in arterial remodelling. Despite the above-described limitations, this may be achievable by several means. For example, the low opacity of optically-cleared arteries might suggest that colorimetric reporters, such as β-galactosidase, that can be visualised by transmission imaging, might be more suitable than fluorescent probes for this purpose. Alternatively, we have generated preliminary data (Bezuidenhout *et al.*, unpublished) to suggest that OPT can be used to identify nanoparticulate within arterial lesions, perhaps providing the means to track phagocytic cells. Finally, whilst the data presented here describe only studies of the mouse vasculature, preliminary studies suggest that is also suitable for imaging morphology and atherosclerotic lesion formation in larger vessels including rat and rabbit aortas (Bezuidenhout *et al*., unpublished). OPT, should, therefore, be equally suited to the study of comparably sized human vessels.

In summary, OPT imaging is a valuable tool for the 3-dimensional evaluation of neointimal and atherosclerotic lesion burden and distribution in mouse arteries; a considerable advance because traditional 2-dimensional methods for the analysis of these lesions are labour intensive and do not effectively represent total lesion volume. Imaging by OPT is relatively fast and high throughput, convenient and non-destructive. Further, in contrast to previously published studies of OPT in adult mouse tissues[19], these methods are achievable using commercially-available equipment and software. This technique should provide the much-needed means to allow 3-dimensional evaluation of morphology to become a routine component of vascular remodelling studies.

## Supporting Information

Figure S1 **Tomographic cross-sections of an OPT-imaged, ligation-injured mouse femoral artery.** After tomographic reconstruction, cross-sectional OPT images (excitation 425 nm with 40 nm band-pass; emission 475 nm long pass) can be animated for qualitative evaluation of vascular structure (in addition to quantitative measurement). In ligation-injured femoral arteries, presented in this way, morphologically-normal vessel can be seen proximal to the site of ligation and the media and lumen can be distinguished. Closer to the ligation, neointima formation is apparent, and this can also be distinguished from the media and lumen. The size of this lesion increases with proximity to the ligation, and eventually completely occludes the vessel. The position of each cross-section within the artery is indicated on the non-tomographic image inset left.
(MOV)

Figure S2 **Tomographic cross-sections of an OPT-imaged, atherosclerotic aortic arch.** After tomographic reconstruction, cross-sectional OPT images (excitation 425 nm with 40 nm band-pass; emission 475 nm long pass) can be animated for qualitative evaluation of vascular structure (in addition to quantitative measurement). In atherosclerotic aortic arches, presented in this way, the media and lumen of the ascending and descending aorta can be seen clearly and some lesion formation is apparent on the lesser curvature of the arch. This can be clearly distinguished from the vessel media and lumen. More severe lesion formation is present at the roots of right carotid and subclavian arteries, and throughout the length of the brachiocephalic artery. The position of each cross-section within the artery is indicated on the non-tomographic image inset left.
(MOV)

Figure S3 **Volume rendering of a tomographically reconstructed, OPT-imaged, atherosclerotic aortic arch.** After tomographic reconstruction, cross-sectional OPT images (excitation 425 nm with 40 nm band-pass; emission 475 nm long pass) can be volume rendered to visualise vascular structure and lesion formation in 3-dimensions. In atherosclerotic aortic arches, presented in this way (a), lesion formation can be observed with the predicated anatomical distribution (brachiocephalic artery, roots of the right carotid and subclavian arteries and lesser curvature of the arch). Regions within cross-sections corresponding to lesion can be segmented, separately rendered, and superimposed on original data to further highlight distribution and extent of lesion formation (b; lesion displayed in red). This process is analogous to that required to quantify lesion volume.
(MOV)

Methods S1 **Protocol for OPT imaging and quantification of murine intimal and atherosclerotic lesions.** Step-by-step protocol detailing procedures for OPT-based examination of intimal and atherosclerotic lesions in mouse arteries.
(DOC)

Methods S2 **Additional methodological detail.** Comprehensive description of the methodology used for all the techniques utilised in these studies.
(DOC)
