# Supplementary material for: Quantitative 3-Dimensional Imaging of Murine Neointimal and Atherosclerotic Lesions by Optical Projection Tomography
Source: PLoS One. 2011 Feb 17;6(2):e16906. doi: 10.1371/journal.pone.0016906 (PMC3040742; doi:10.1371/journal.pone.0016906)
Supplement: Methods S1 — Protocol for OPT imaging and quantification of murine intimal and atherosclerotic lesions. Step-by-step protocol detailing procedures for OPT-based examination of intimal and atherosclerotic lesions in mouse arteries. (DOC) [file pone.0016906.s004.doc]

**Supplementary Protocol**

Step-by-step protocol for the preparation, imaging, reconstruction and quantification of neointimal and atherosclerotic lesions in mouse arteries by optical projection tomography (OPT).

*Sample Preparation & Imaging*

1. Perfusion fix arteries *in vivo*, under terminal anaesthesia using >20ml PBS followed by >20ml 10% buffered formalin. This is essential to maintain luminal conformation, and because the strength of fluorescent emission from any remaining blood in the vessel lumen compromises the dynamic range available for imaging of the vessel wall. **Time required: ~15 mins per mouse.**
2. Isolate arteries of interest, removing as much peri-adventitial material as possible. Post-fix in 10% buffered formalin overnight, before storage in 70% ethanol until needed. **Time required: overnight.**
3. Individually embed arteries in 1.5% low melting point agarose (Invitrogen, UK), pre-filtered through Whatman 113V paper (GE Healthcare, UK). Attach each to a magnetic OPT mount (Bioptonics, UK) with cyanoacrylate adhesive so that the vessel axis is in line with that of the mount. Trim excess agarose to a conical shape. Dehydrate in 100% methanol for at least 12 hours – vessels can be stored indefinitely at this stage. **Time required: 20 mins preparation per sample, dehydration overnight.**
4. 12-24 hours prior to scanning, optically clear vessels by immersion in a mixture of 1 part benzyl alcohol to 2 parts benzyl benzoate. **Time required: overnight.**
5. Install cleared, mounted vessels in a calibrated, Bioptonics 3001 tomograph (Bioptonics, UK). Set resolution to 1024x1024, and determine an optical magnification that allows the entire area of interest to be seen. In our experience, this should be 6µm for ligation-injured femoral arteries, 4µm for wire-injured femoral arteries, and 5.5µm for atherosclerotic aortic arches. Adjust sample position so that it rotates upon its own axis in the centre of the field of view in the bright-field, transmission channel. For arteries in which lesion formation is anticipated over a long length of vessel (relative to vessel diameter), such as the wire-injured femoral artery, image quality can be dramatically improved by taking 2 or more, higher magnification scans at several vertical positions.
6. In the GFP1 filter emission channel (excitation filter 425nm with 40nm band-pass; emission filter: 475nm long pass), focus specimen and adjust exposure time so as to maximise the dynamic range of the resulting image. Care should be taken to avoid over-saturation at any increment of rotation – this will compromise later re-construction. Scan vessel in GFP1 emission channel only, with a 0.9º rotation step. **Time required: 20 mins per scan.**
7. At completion, confirm success and quality of data acquisition using DataViewer software (Skyscan, Belgium). Remove specimen from scanner. If subsequent histological analysis is intended, place sample in 100% methanol for >24 hours before processing to paraffin wax as normal.

*Reconstruction & Quantification*

1. Tomographic re-construction by filtered back-projection is performed using NRecon software (Skyscan, Belgium) or similar. Misalignment compensation is determined by scrutiny of test reconstructions performed about a pre-determined value (11 x 0.5 steps). Image intensity levels can be adjusted to improve clarity (again, over-saturation must be avoided). Full ring artefact reduction should be applied. After completion, the quality of reconstruction should be manually verified using DataViewer software (Skyscan, Belgium). Reconstructions can be performed unattended, in batches. **Time required: ~10 mins setup per sample, ~2 hours for re-construction.**
2. Lesion and lumen volumes/profiles are determined using CTan software (Skyscan, Belgium). A vertical region of interest is first defined to encompass the length of vessel in which lesion formation is present. If luminal dimensions are to be recorded, the length of this region must be constant between studied vessels.
3. Within this length, for neointimal lesions, the position of the internal elastic lamina (i.e. internal border of the media) must be manually defined. For atherosclerotic lesions with intra-plaque lipid pools, it is the plaque outline that must be defined. In both cases, this is achieved by tracing the appropriate border, for 1 in every 50 re-constructed cross-sections. Positions in the interleaved cross-sections are interpolated by software and should be verified manually and adjusted where necessary.
4. This 3-dimensionally defined volume must next be further segmented according to a grey-level threshold, which should be adjusted so that only lesion is selected. From these definitions, measurements can be output. These include total lesion volume (object volume), luminal volume (total volume – object volume) and the distribution of lesion and lumen cross-sectional area along the axial length of the studied vessel. **Time required: 10-30 mins per sample.**
