# Supplementary material for: Quantitative 3-Dimensional Imaging of Murine Neointimal and Atherosclerotic Lesions by Optical Projection Tomography
Source: PLoS One. 2011 Feb 17;6(2):e16906. doi: 10.1371/journal.pone.0016906 (PMC3040742; doi:10.1371/journal.pone.0016906)
Supplement: Methods S2 — Additional methodological detail. Comprehensive description of the methodology used for all the techniques utilised in these studies. (DOC) [file pone.0016906.s005.doc]

**Supplementary Methods**

*Induction of neointima formation*

Acute vascular injury surgery was performed in male, 12 week old C57Bl6/J mice (Harlan, UK). Wire-injury to the femoral artery was performed essentially as described by {Sata, 2000 #406}. Briefly, under isoflurane anaesthesia, the left femoral artery was exposed and isolated from the femoral nerve about the femeropopliteal bifurcation. Through a transverse arteriotomy in the popliteal branch, a 0.014” diameter straight-sprung wire was inserted, advanced into the femoral artery and allowed to dilate it for 1 minute. Upon its withdrawal the popliteal artery was ligated just proximal the arteriotomy and reperfusion of the femoral artery observed. Ligation-injury was performed to the right femoral artery of same mice, in a manner similar to that described by {Kumar, 1997 #470} for the carotid artery. As for wire-injury, the femoral artery was exposed and isolated from the adjacent vein. A 5-0 silk suture was tied across the femeropopliteal bifurcation. Animals were allowed to recover for 28 days before sacrifice by transcardiac perfusion fixation and exsanguination under terminal anaesthesia. Left and right femoral arteries were isolated and cleaned of extraneous peri-adventitial material.

*Induction of atherosclerosis*

Atherosclerotic lesion formation was induced in male, 6 week old ApoE-defieicnt mice (bred in house) by feeding of a western diet containing 0.2% cholesterol (Research Diets, New Jersey, USA) for 12 weeks. At the end of this period, animals were killed by perfusion fixation, as above. The arterial tree was carefully dissected and the aortic arch further isolated along with its major branches (brachiocehpalic artery, left common carotid and subclavian arteries).

*Optical tomographic scanning and quantification*

For three-dimensional evaluation of femoral arteries and aortic arches, vessels were embedded in 1.5% low melting point agarose for analysis. Specimens were dehydrated in 100% methanol and optically cleared in a refractive index matching solution of benzyl alcohol and benzyl benzoate (1:2), each for 24 hours. Vessels were imaged using a Bioptonics 3001 OPT tomograph. All studies on injured arteries were performed using emission imaging, after UV illumination (425nm excitation filter with 40nm band pass; 475nm long pass emission filter; 1.048Mpixel scanning resolution). For each vessel type, a magnification was chosen to provide the smallest voxel size whilst allowing the entire region of interest to be covered. This resulted in voxel sizes of 216, 64 and 166μm^3^ respectively for wire- and ligation-injured femoral arteries and atherosclerotic aortic arches. For each vessel, exposure time was adjusted to maximise the dynamic range of the resulting image and was approximately 400, 800 and 1000ms per projection for wire- and ligation-injured femoral arteries and atherosclerotic aortic arches, respectively. Raw data (400 projections per scan at 0.9**°** increments) was subject to Hamming-filtered back-projection using NRecon software (Skyscan, Belgium). Quantification was performed using CTan software (Skyscan, Belgium; see **Supplementary Protocol**). Briefly, in injured femoral arteries, lesion and lumen volumes were segmented by semi-automated tracing of the estimated position of the internal elastic lamina and subsequent grey-level thresholding within this to delineate neointima from lumen. In atherosclerotic aortic arches, plaque volume was segmented by semi-automated tracing the entire lesion border.

*Histological examination*

After tomographic scanning, vessels were immersed in methanol for 24 hours to remove the refractive index matching solution, and trimmed of excess agarose before infiltration with, and embedding in, paraffin wax. 3-4μm thick serial sections were cut perpendicular to the vessel axis. For atherosclerotic aortic arches, only the brachiocehpalic trunk was sectioned. Histological sections corresponding to OPT re-constructed slices were identified and stained using the ‘United States Trichrome’ (UST) method to highlight vessel morphology {Hadoke, 1995 #474}, and adjacent sections with picro-sirius red to determine collagen content. Planimetric measurements of lesion size (defined as the area delineated by the luminal border and internal elastic lamina) were recorded from photomicrographs of UST-stained sections using Photoshop CS4 Extended software (Abobe Systems Inc, USA).

*Immunohistochemistry*

Immunohistochemistry was performed according to the labelled streptavidin-biotin (LSAB) method. Briefly, for αSMA (α-smooth muscle actin) immunohistochemistry, paraffin sections were re-hydrated and non-specific binding sites blocked by application of 2.5% bovine serum albumin (BSA), 2.5% milk power in PBS (αSMA immunohistochemistry only) followed by 20% goat serum. Sections were incubated with mouse anti-mouse αSMA (Sigma, UK; 1/400 dilution; 30 mins) primary antibody before extensive washing and application of biotinylated goat anti-mouse IgG (Vector Labs, UK; 1/400 dilution; 30 mins) secondary antibody. After further washing, sections were incubated with streptavidin-conjugated horseradish peroxidase (Extravidin-Peroxidase; Sigma, UK; 1/400 dilution; 30 mins). Immunoperoxidase complexes were visualised by the subsequent application of 3,3-diaminobenzidine (DAB+; Dako Labs, UK) and its development into an insoluble product. Mac2 immunohistochemistry was performed in a similar manner, using only 20% goat serum to block non-specific binding, with rat anti-mouse Mac2 (Cederlane Inc, USA; 1/6000 dilution; overnight) as primary antibody and with biotinylated goat anti-rat IgG (1/200 dilution; 30 mins) as secondary antibody.

*Statistics and data analysis*

Correlation between histology- and OPT-derived planimetric measurements of lesion size was performed by linear regression. The deviation of the slope of this relationship from the hypothetical value m=1.0 was determined by F-test. Bland-Altman charts were producing by plotting the difference in lesion area as determined from histology and OPT images against their average, for each measurement pair. Profiles of lesion distribution were produced by plotting 2-dimensional lesion and lumen cross-sectional area against distance from a fiduciary reference (e.g. the femoropopliteal bifurcation). For clarity, in such profiles, each data point represents the mean of 20 re-constructed slices (80µM for ligation-injured vessels, 120µM for wire-injured vessels).
